# HotSwap for bioinformatics: A STRAP tutorial

**DOI:** 10.1186/1471-2105-7-64

**Published:** 2006-02-09

**Authors:** Christoph Gille, Peter N Robinson

**Affiliations:** 1 Institute for Biochemistry, Charité University Hospital, Humboldt University Berlin, Germany; 2 Institute of Medical Genetics, Charité University Hospital, Humboldt University Berlin, Germany

## Abstract

**Background:**

Bioinformatics applications are now routinely used to analyze large amounts of data. Application development often requires many cycles of optimization, compiling, and testing. Repeatedly loading large datasets can significantly slow down the development process. We have incorporated HotSwap functionality into the protein workbench STRAP, allowing developers to create plugins using the Java HotSwap technique.

**Results:**

Users can load multiple protein sequences or structures into the main STRAP user interface, and simultaneously develop plugins using an editor of their choice such as Emacs. Saving changes to the Java file causes STRAP to recompile the plugin and automatically update its user interface without requiring recompilation of STRAP or reloading of protein data. This article presents a tutorial on how to develop HotSwap plugins. STRAP is available at  and .

**Conclusion:**

HotSwap is a useful and time-saving technique for bioinformatics developers. HotSwap can be used to efficiently develop bioinformatics applications that require loading large amounts of data into memory.

## Background

Bioinformatics applications now routinely analyze large amounts of data. Development of programs thus often involves repeated loading and testing of megabytes or more of data to check program correctness or to optimize algorithms and parameter settings. Compiled languages such as C generally offer a much quicker execution time than comparable programs written in scripting languages, but each new compilation can mean loading and initializing the data into the program being developed, which can significantly prolong development time. In general, it is not possible to change program code at runtime.

Java is a compiled language that produces platform-independent byte code that runs on a fast virtual machine on nearly all modern architectures. The execution speed is similar to that of the languages C or C++. The Java HotSwap technique provides a mechanism that allows developers to keep program code and data in memory during recompilation and thereby significantly accelerate and simplify development of bioinformatics applications that are intended to analyze large amounts of data. HotSwap provides a dynamic class redefinition capability for the Java virtual machine (JVM) that allows developers to change a class inside the JVM at runtime [1,2]. This allows developers to make changes at run time that they would otherwise make off-line with subsequent recompilation. This can significantly reduce development turnaround time.

STRAP is a versatile workbench for protein analysis that can be used to generate and refine multiple alignments, to download PDB files from public ftp servers, visualize protein structural data with plugin or integrated protein structure viewers, and to map mutations onto three dimensional protein structures [3-5]. STRAP allows users to develop their own plugins that take advantage of the STRAP infrastructure; that is, users do not need to write code to load PDB files, to visualize alignments, to translate nucleotides, but rather can concentrate on the logic of the problem they would like to solve.

We have recently extended the capabilities of STRAP to allow developers to use the Java HotSwap technique to develop novel bioinformatics programs, either as plugins for STRAP or as prototypes for independent applications. We have extended the Inxar HotSwap implementation [6] so that it is now additionally possible to use Java inner classes. The techniques described in this work could also be applied to other Java analysis tools for bioinformatics. In the following, we provide a tutorial with a realistic application to demonstrate the usefulness of HotSwap in the development of bioinformatics applications.

## Implementation

STRAP is implemented in the Java programming language [7]. Users can develop their own plugins to perform specific types of analysis and also take advantage of the other features of the STRAP workbench, including online retrieval of sequence and structure data, sequence alignments, and structure visualization. We based our implementation of HotSwap on an open-source project of Inxar [6] and added code to interact with STRAP.

Inner classes are a useful feature of Java since version 1.1. We extended HotSwap support to inner classes, such that the "swapped" classes can now contain inner classes. The complete source code for HotSwap as well as the rest of STRAP is freely available.

HotSwap can be used for all types of STRAP plugin. Even if implemented interfaces are added or removed, and thus changes are made to the public signature of the plugin, no recompilation is needed. The HotSwap mechanism has been implemented only for plugins, and any changes that developers might make to the core STRAP classes will require recompilation. The code used for implementing HotSwap in STRAP can be used to implement HotSwap in other applications. A complete example is available from the homepage of the authors [8].

## HotSwap for STRAP: A tutorial

In the following, we show how to develop a relatively simple, but useful plugin designed to predict coiled coil regions in protein sequences. The development of such plugins often involves many cycles of incremental changes to the code. Normally, this would require recompilation of the code for the entire program. Using STRAP's HotSwap capabilities, developers can load multiple protein sequences into STRAP graphical user interface (GUI), display the predicted sequences by highlighting the predicted sequence regions in the GUI, and make changes to the code of the plugin. The hotswap facility recognizes and recompiles the modified plugin source code and "swaps " the new objects back into the main STRAP program. Reloading the protein sequences or restarting STRAP is not required. Any changes in the predictions of the plugin are automatically displayed in the STRAP GUI.

### Coiled coil prediction

As an example, we have adapted a previously published program for identification of coiled coils in protein sequences [9]. Coiled coils are helical bundles of 2–5 *α *helices with a distinctive packing of amino acid sidechains at the helix-helix interfaces called "knobs-into-holes ". Coiled coils are widespread protein motifs that form large, mechanically rigid structures such as hair (keratin), blood clots (fibrin), and extracellular matrices (laminin), and also frequently provide oligomerization domains (leucine zippers) [10,11]. The structure of the helix-helix interfaces results in every seventh residue occupying an equivalent position on the helix surface, so that the sequences of coiled-coil proteins display a heptad periodicity in the chemical nature of their sidechains, which is a main feature recognized by coiled-coil prediction programs [12].

### STRAP plugins

STRAP provides standardized interfaces to its many functions and windows. This simplifies the development of plugins, because users only need to implement a few methods in order to create code that interacts with STRAP. The first step in creating a new plugin consists in choosing one of the presupplied STRAP code skeletons (Java interfaces) (Table 1). Developers should be able to choose a plugin skeleton that supports the data exchange and visualization functionality needed by their application.

**Table 1:** STRAP HotSwap Plugin Types. This table gives an overview of some of the plugins that are already available in STRAP and can be used as examples for development new plugins. STRAP plugins are defined by a Java interface with functions that users can implement in order to have their plugin interact with STRAP. *Protein Protein Distance *returns values for all pairs of proteins in a set of proteins. The values can be used for distance matrices. *Sequence Aligner *is used as a wrapper around programs such as clustalW to perform multiple alignments and display them in the STRAP GUI. Likewise *Protein Viewer *is a wrapper around external protein viewers such as rasmol or pymol. *Superimpose3D *is an interface for methods that superimpose one protein structure onto another by calculating a 3 × 3 rotation matrix and a {dx, dy, dz} translation matrix. *Value Of Residue *is an interface for methods that return a float value for each residue of a protein in order to plot a profile along the sequence of the protein. *Value Of Protein *is for plugins that calculate a float value for individual proteins; the STRAP GUI will display these values as a bar chart. Fuller documentation of these and other plugin types is available online. Each example plugin is already included in the main STRAP distribution, and source code can be seen from within the STRAP GUI. The last column explains the purpose of the most important methods of the respective plugins. In general, these methods are designed to interact with the rest of the STRAP GUI to display the results of the plugin's analysis. See text for further details.

**Plugin Type**	**Example**	**Methods in Interface**
*SoR*	CoiledCoil	See text

*Protein Protein Distance*	SequenceDisSimilarity-AsAligned	• **get Value **Returns a real number representing the degree of dissimilarity between two or more proteins. In the example class, it returns the proportion of aligned residues that are identical. This information is then used by the STRAP GUI to display a graph or table with the results.

*Sequence Aligner*	A variety of well-known algorithms are implemented as plugins, for example "Multiple AlignerClustalW"	• **Get AlignedSequences **Returns the results of the alignment algorithm for display in the STRAP GUI.

*Protein Viewer*	Interfaces to rasmol, pymol, others	• **getProtein **Returns a reference to the associated STRAP protein object. • **getSelectedAminoAcids **Causes selected residues to be shown as selected by the protein viewer.

*Superimpose3D*	SuperimposeGoede	• **getRotation **Returns a 3 × 3 rotation matrix. • **getTranslation **Returns a {dx, dy, dz} translation vector.

*Value Of Residue*	Solvent Accessibility	• **get Values **Returns the values for each residue which are then displayed by the STRAP GUI as a profile.

*Value Of Protein*	countResidues	• **get Value **Returns a value for the protein which is then displayed by the STRAP GUI as a bar in a bar chart.

To choose a plugin type, users switch to STRAP's Java plugin menu and switch to expert mode. STRAP creates a new plugin if users mark the interface node that represent the desired plugin type and click on the "new plugin" icon. STRAP generates a skeleton class that implements the desired interface as well as other interfaces and methods that provide additional functionality such as event listening or control panels. To see what methods need to be implemented for a particular plugin type, the users activate the tree node of the interface and then can click on the Java Source Code icon to view the code of the Java interface. The generated class provides skeleton implementations of all required methods so that the program already compiles and can be executed by pressing the "apply or start plugin" button. Users can now add the application-specific code.

### SoR plugins: selection of residues

In the present example, we chose the SoR interface because this plugin allows simple visualization of **S**elections **o**f **R**esidues. In our case, we will write code to predict the presence of coiled coils in a protein sequence and will use the SoR plugin type to visualize the coiled coils in the amino acid sequence and in the corresponding three-dimensional protein structure (if structure data is available for the protein in question).

Once the user has pressed the "new plugin" button STRAP provides skeleton code for the interface, including the methods setProtein, getProtein, getSelectedAminoacids, and setSelectedAminoacids. Depending on the application, users will need to change this code to get the required functionality. Furthermore, users need to define their own methods to interact with these methods. In the present example, we need to implement methods for coiled-coil prediction; these methods need to get protein sequences from STRAP and to return the results of analysis in the form of an array of true or false values. We have named the new plugin class CoiledCoil. The source code, CoiledCoil.java, is available from the STRAP website.

### set protein

This method is called automatically by the STRAP GUI to communicate the protein object to the plugin immediately after the instance of the plugin class is created. Note that one instance of the plugin is created for each protein that is selected in the STRAP GUI, so that user code in the plugin analyzes only a single protein. STRAP causes one instance of the plugin to be executed for each protein selected, so that multiple proteins can be analyzed.

User code can take advantage of the attributes and methods of StrapProtein objects. For instance, to get the codon (triplet) coding for an amino acid, call

byte [3] = getResidueTriplet (idx, new byte [3])

, where idx is an integer value representing the position of the amino acid in the protein chain. The three bases are returned in an byte array. To avoid inefficiency due to the frequently invoked method getResidueTriplet repeatedly creating byte arrays, the calling code must provide memory space of three bytes with the second method argument. Thus the byte array can be created once and used many times, which significantly improves performance.

As another example,

float z = getResidueCalphaZ (idx)

is used to get the z coordinate of the *C*_*α *_atom of the residue at position idx. If no coordinates are loaded for the respective protein or the coordinates at the given index are not resolved in the structure, NaN is returned. Javadoc documentation of all methods and fields for the class StrapProtein and other STRAP classes may be found at the project website [13].

Note that since StrapProtein objects can be created from PDB files, FASTA files, or translated Genbank nucleotide files, user code should not assume that all attributes of a StrapProtein object will have values; for instance, if a StrapProtein object was constructed from a FASTA file, only attributes related to the amino acid sequence will have values, and calls to other methods such as getResidueSolventAccessibility will return NULL.

In CoiledCoil.java, we have declared a member variable private StrapProtein protein. The method public void setProtein( ) is called immediately after object creation and initializes protein as one of the proteins selected in the STRAP graphical user interface (GUI).

getProtein( ) is the counterpart to setProtein. Its task is to return a reference to the protein to the STRAP GUI. Each instance of SoR refers exactly to one protein.

### getSelectedAminoacids

The purpose of getSelectedAminoAcids is to return an array of boolean values. true at a given index indicates that the corresponding amino acid residue is "selected" according to the logic of the plugin (in our case, it indicates that the residue is part of a predicted coiled coil).

**Figure 1:**
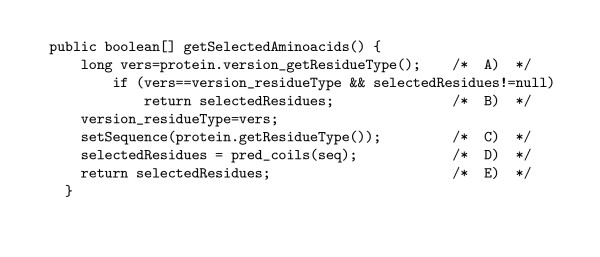
Java code for the method getSelectedAminoacids( ). **A**. Initialize the local variable vers with the current version of the amino acid sequence. This value is changed whenever the residue sequence changes. Before the analysis is performed, the value of selectedResidues is null, so that the code following these lines will always be executed when the plugin is initialized; **B**. If the residues have not been changed since the last analysis, just return the previously selected residues; **C**. We use the private class method setSequence to alter the class variable seq, which holds the current amino acid sequence. The method getResidueType of the class StrapProtein is used to retrieve the current amino acid sequence from the STRAP GUI; **D**. pred_coils performs the analysis for coiled coil regions, and places the results of its analysis in the array selectedResidues; **E**. Finally, we return the array selectedResidues to the STRAP GUI, which uses this information in order to mark the residues in amino acid sequences or protein structures.

Figure 1 shows the Java code for the method get SelectedAminoacids( ), and is typical of the way users can connected their own code to STRAP. get SelectedAminoacids( ) calls the method pred_coils ( ), which performs the actual prediction of coiled coil regions. The code of pred_coils is closely based on the C program ncoils [9]. The method pred_coils calculates a value for each residue and compares it to a threshold value for being in a coiled coil region. If the value is above the threshold, the corresponding position of selectedResidues is set to true:

if (P[i]>=min_P) selectedResidues [i]=true;

Before the calculation is started, the method checks whether a previously calculated result can be returned instead. This is the case if and only if the method was called previously with exactly the same amino acid sequence. The amino acid sequence of the protein may be changed. For example, users can change the amino-acid sequence directly. If the protein object was created from genomic DNA sequence, altering the indicated intron-exon boundaries will also change the predicted amino acid sequence, and this will be reflected by corresponding changes in the SoR instance. To decide whether the amino acid sequence has been changed the version number can be requested from the protein object using the method long version_residueType ( ).

### setSelectedAminoacids

STRAP has a button to add or remove a single residue under the cursor to or from a selection. This feature is designed for certain selections such as selections in 3D-viewers. For the class CoiledCoil this feature is not required and the method body is kept empty.

### Listening to events in STRAP

The interface StrapListener is required for plugins which need to be informed when data changes. For instance, if the user changes the amino acid sequence of a protein in the STRAP GUI, the plugin may need to be informed in order to recalculate its analysis. In general, STRAP creates a separate instance of each plugin class for each protein being analyzed. To improve efficiency of the GUI, STRAP creates one instance of the plugin just for the purpose of listening for events that receives a message from the STRAP GUI each time the sequence of one of the proteins is changed.

The appropriate answer to this message would be to tell STRAP and all listeners that the residue selection might have changed. As a consequence STRAP would redraw all alignment windows and by doing so would request the selected residues by calling get SelectedResidues ( ) of the CoiledCoil instances. The instance of CoiledCoil of the changed protein would be forced to recompute the prediction. The instances of the other proteins that did not change would quickly return the previously calculated prediction. However, since STRAP must redraw the alignment anyway when the residue sequence changes, the interface StrapListener was omitted in CoiledCoil.

### Catching errors

If an exception is thrown, the stack trace can be examined by opening the debugging panel in the STRAP GUI (Options menu, "finding errors").

## Discussion

STRAP provides a number of plugin types that can be extended by developers to perform specific kinds of analysis. In each case, Java interfaces are used to define methods required for interaction with the STRAP GUI, which generally involves getting references to single or multiple protein objects, and returning the results of analysis in a way that can be displayed by the STRAP GUI. For instance, the plugin type ValueOfProtein can be used to assign numerical scores for proteins (according to a user-defined analysis) and to display the results of analysis as bars in the row header of the multiple sequence alignment. The essential method that needs to be implemented by the plugin developer is get Value ( ), which returns a numeric value representing the results of the analysis of the protein represented by the plugin instance. Further examples and documentation are available at the project website.

The most important classes to learn in order to create useful plugins are *Strap Protein *(which contains all data of a protein such as the amino acid sequence, the name, the nucleotide sequence, the secondary structure and the coordinates), *Strap Event *(An instance of this class is broadcasted whenever changes occur and provides methods to request the type of event and the event source), and *Strap Align *(which is the root component and contains all information of the alignment. It is used by plugins to request all proteins or all selected proteins.)

### HotSwap for bioinformatics

The full value of the HotSwap technique is apparent when users are developing a new plugin, which often means that various parts of the program code are repeatedly changed during program debugging, testing, and optimization. To provide a first impression of the usefulness of this technique, we suggest that interested readers load the code for the CoiledCoil plugin from the STRAP website into the plugin folder and import the multiple FASTA file CoiledCoil.fasta, which contains multiple protein sequences, some of which have coiled coil regions. Users can use the plugin as provided to visualize the coiled coil regions, and then open the Java code and make changes to the pred_coil method or to the array HEPTAD_WEIGHT which is used for the analysis. STRAP will automatically recompile CoiledCoil.java, while maintaining all links to the rest of the STRAP GUI. Users can then see any changes in the "prediction" of the plugin immediately, without having to recompile the entire STRAP application or reload the protein sequences. Developers increasingly make use of complex APIs for the development of new applications. STRAP is able to automatically load numerous Java archive (".jar") files on demand and can be extended to load any archive file publically available over the internet. One of the most useful archives at present is the BioJava API [14]. Developers can use any of the classes of BioJava to write STRAP HotSwap plugins. There are two utility classes responsible for conversion between STRAP and BioJava, BiojavaSequence2StrapProtein and StrapProtein2BiojavaSequence. Example BioJava plugins are available in the STRAP GUI and are referenced in the online documentation for BioJava [15].

## Conclusion

We have presented a tutorial on how to use HotSwap to develop plugins for STRAP. HotSwap has been integrated into recent versions of Java [16]. Although HotSwap was originally conceived for applications that must run continuously without any interrupts, such as transaction processors or air flight controllers, it has obvious utility for the development of bioinformatics applications that process large amounts of data and require extensive optimization.

In STRAP, no additional work is required to use HotSwap techniques. The functionality of STRAP allows users to develop plugins that use STRAP's graphical user interface to interact with various protein viewers, to translated nucleotide sequences, to download files from a variety of servers, among many others, so that users can concentrate on the logic of the analysis they would like to develop rather than on the framework.

## Availability and requirements

Project Name

STRAP

Project Homepage





Operating Systems

Platform independent. STRAP is a Java application that requires at least Java 1.4 to run. It can be run as a WebStart application or downloaded and started locally. STRAP has been tested under linux, Windows and MacOS X.

Programming language

Java. STRAP is open source, and the source code can be accessed directly from the STRAP GUI or with the file strap.jar, which is available for download without restrictions. Some of the embedded applications in strap are written in C/C++.

License

GNU General Public License.

Any restrictions to use by non-academics

There are no restrictions concerning the core STRAP code. However, a few of the embedded applications do have restrictions. Restricted applications are marked in the STRAP GUI, and non-academics are advised to consult the webpages of the respective applications to determine the restrictions on use.

## Authors' contributions

CG integrated HotSwap functionality into the STRAP protein workbench. PNR developed the CoiledCoil plugin and wrote the manuscript. Both authors tested HotSwap plugins, read, and approved the final manuscript.

## References

[B1] Dmitriev M (2001). Safe Class and Data Evolution in Large and Long-Lived Java Applications. Tech Rep Sun Technical Report SMLI TR-2001-98.

[B2] Dmitriev M (2001). Towards Flexible and Safe Technology for Runtime Evolution of Java Language Applications. OOPSLA 2001 International Conference.

[B3] Gille C, Frommel C (2001). STRAP: editor for STRuctural Alignments of Proteins. Bioinformatics.

[B4] Gille C, Lorenzen S, Michalsky E, Frommel C (2003). KISS for STRAP: user extensions for a protein alignment editor. Bioinformatics.

[B5] Gille C (2006). Structural interpretation of mutations and SNPs using STRAP-NT. Protein Sci.

[B6] Inxar HotSwap. http://directory.fsf.org/libs/java/HotSwap.html.

[B7] Java. http://www.java.sun.com.

[B8] Keggano Homepage. http://www.charite.de/bioinf/strap/sysbio/kegganno.

[B9] Lupas A, Van Dyke M, Stock J (1991). Predicting coiled coils from protein sequences. Science.

[B10] Lupas A (1996). Coiled coils: new structures and new functions. Trends Biochem Sci.

[B11] Burkhard P, Stetefeld J, Strelkov SV (2001). Coiled coils: a highly versatile protein folding motif. Trends Cell Biol.

[B12] Lupas A (1997). Predicting coiled-coil regions in proteins. Curr Opin Struct Biol.

[B13] STRAP website. http://strapjava.de.

[B14] Mangalam H (2002). The Bio* toolkits-a brief overview. Brief Bioinform.

[B15] BioJava cookbook, online documentation. http://www.biojava.org/docs/bj_in_anger/index.htm.

[B16] See for instance. http://java.sun.com/j2se/1.4.2/docs/guide/jpda/enhancements.html.

